# Coming in handy: *CeTI-Age* — A comprehensive database of kinematic hand movements across the lifespan

**DOI:** 10.1038/s41597-023-02738-3

**Published:** 2023-11-25

**Authors:** Evelyn Muschter, Jonas Schulz, Máté Tömösközi, Leonie Herbst, Lena Küssel, Merve Sefunç, Stefan Holtzhausen, Stefanie Speidel, Frank H. P. Fitzek, Shu-Chen Li

**Affiliations:** 1 Technische Universität Dresden, Centre for Tactile Internet with Human-in-the-Loop, Dresden, 01062 Germany; 2 Technische Universität Dresden, Chair of Lifespan Developmental Neuroscience, Dresden, 01062 Germany; 3 Technische Universität Dresden, Deutsche Telekom Chair of Communication Networks, Dresden, 01062 Germany; 4 Mimetik UG, Dresden, 01219 Germany; 5 Technische Universität Dresden, Chair of Virtual Product Development, Dresden, 01062 Germany; 6 National Center for Tumor Diseases (NCT/UCC) Dresden, Department of Translational Surgical Oncology, Dresden, 01307 Germany

**Keywords:** Scientific data, Ageing

## Abstract

The Tactile Internet aims to advance human-human and human-machine interactions that also utilize hand movements in real, digitized, and remote environments. Attention to elderly generations is necessary to make the Tactile Internet age inclusive. We present the first age-representative kinematic database consisting of various hand gesturing and grasping movements at individualized paces, thus capturing naturalistic movements. We make this comprehensive database of kinematic hand movements across the adult lifespan (*CeTI-Age-Kinematic-Hand*) publicly available to facilitate a deeper understanding of intra-individual–focusing especially on age-related differences–and inter-individual variability in hand kinematics. The core of the database contains participants’ hand kinematics recorded with wearable resistive bend sensors, individual static 3D hand models, and all instructional videos used during the data acquisition. Sixty-three participants ranging from age 20 to 80 years performed six repetitions of 40 different naturalistic hand movements at individual paces. This unique database with data recorded from an adult lifespan sample can be used to advance machine-learning approaches in hand kinematic modeling and movement prediction for age-inclusive applications.

## Background & Summary

Hand movements feature prominent functions for humans to interact with the environment and to communicate with others. In recent years, humans not only interact with their hands in the real world, but increasingly in different types of digitized multimedia environments, such as virtual or augmented reality^1–3^. Since hand movements serve as a crucial interaction interface, to make use of them in any of such scenarios as well as in digitally transmitted remote human-machine or human-human interactions through the Tactile Internet (TI)^4^, hand kinematics need to be well tracked^5,6^ and in some cases modeled^7–9^, or predicted^10–12^. Although several useful databases of hand movements^13–17^ exist, most come with certain limitations. The present database aims to improve on several of these aspects.

First, in order to increase generalizability to a broader population of users, the data should be representative particularly regarding age inclusion^18^. To this end, sensor data of various hand movements should be recorded from potential user samples covering a sufficiently wide range of the adult lifespan. Covering data also from middle-aged and older adults is important because aging research shows that brain aging contributes to age-related impairments in executing and perceiving sensorimotor movements^19^. Specifically, age-comparative studies found slower movement time^20,21^, reduced strength, dexterity and sensation^22^, as well as reduced movement precision and independence of finger movements^23^, smaller grasp aperture^21^, and lower movement stability during reaching^24^ in older compared to young adults. Furthermore, there are indications that these age-related differences observed in real settings may also carry over into virtual environments^25^. With rapid population aging worldwide^26^, age-adjusted designs of digital devices and software for applications of human-machine or human-human interactions in virtual and remote environments would be crucial^18^. Lastly, although about 90% of the population worldwide are right-handed^27,28^ and perform manual tasks faster and more precisely with the right hand, data from left-handed participants should also be included^16^ to not neglect applications for left-handed individuals.

**Fig. 1:**
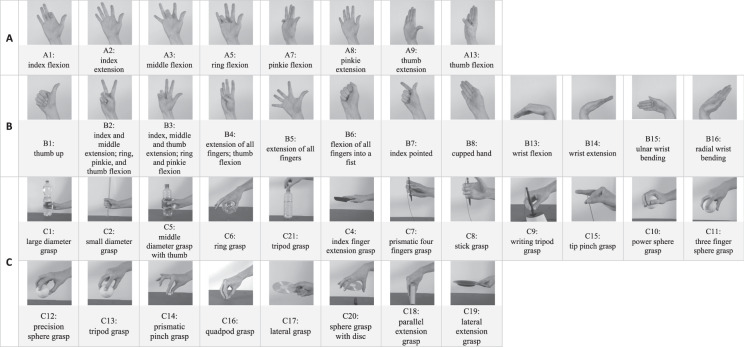
Visual representation and actionID of each movement in three different categories: basic finger movements (**A**), hand postures and wrist movements (**B**), and grasping and functional movements (**C**).

Second, in order to map a wide array of hand movements, it is crucial to include basic finger movements, hand postures and wrist movements, as well as different grasp and functional movements. Here, the extensive Ninapro Project database^13,17^ is notable. Importantly, cross-referencing the grasp movements with established grasp taxonomies^29,30^ allows for proper cross-validation and ensures the inclusion of a broad range of different grasp types, including distal, cylindrical, spherical, and ring grasps^30^. Figure 1 depicts the selection of all hand movements included in the present work (the movement naming convention of the Ninapro Project database was maintained^13^ for consistency across databases).

To improve performance accuracy of machine-learning algorithms for recognizing, classifying and predicting natural hand movements, we adapted the acquisition protocol in terms of movement instructions. Previous work confirmed that videos is an adequate modality for instructing hand movements^31^. However, in order to capture potential intra-individual and inter-individual differences in movements, participants in our database naturally performed each movement in their individual paces after the instruction video was shown, instead of just mimicking the movement in temporal synchrony with the video. Moreover, it would be helpful to also include individual anthropometric measures in the database, which was not available previously. This allows for improved motion analysis, synthesis, and animations. Similarly, this data is helpful in accounting for a potential technical source of data variability in sensor positioning that is associated with individual differences in hand anthropometry. Furthermore, the anthropometric data could be used for other research inquiries, such as ergonomic design of hand-held devices or datagloves, and forensic anthropology.

Taken together, whereas the Ninapro Project database^13,17^ is currently considered a benchmark of hand-movement data, with the *CeTI-Age-Kinematic-Hand* database we provide significant extensions to existing datasets in several aspects, by including (i) the first data from underrepresented members of the general population that cover a broad continuous adult age range from 20 to 80 years, (ii) novel anthropometric measures, (iii) stimulus videos for reproducibility, and (iv) an improved acquisition protocol that facilitates recording of naturalistic individual hand movements. These features are instrumental for developing age-inclusive technologies.

## Methods

### Participants

Data was recorded from sixty-three participants (33 female/ 30 male, *M*_Age_ = 47.8 ± 18.7 years, 6 left-handed) in three continuous age groups, covering the age range from 20 to 80 years (see Table 1 for details on demographic and anthropometric measurements). All participants provided written informed consents and consented to the public sharing of the study data. The study was approved by the Ethical Committee of the Technische Universität Dresden (SR-EK-5012021) and was conducted in accordance with the Declaration of Helsinki. The data was pseudonymized and a unique ID was assigned to each participant. Questionnaire data were collected and managed using the REDCap electronic data capture tools^32,33^ hosted at Technische Universität Dresden.

**Table 1:** Demographic and anthropometric data of the *CeTI-Age-Kinematic-Hand* sample.

	Young adults	Middle-aged adults	Old adults
Age range (years)	20–39	40–59	60–80
N	20	22	21
Sex (*N*_female_/*N*_male_)	10/10	13/9	10/11
Age mean ± SD (years)	24.7 (4.7)	48.7 (6.8)	68.7 (4.3)
Height mean ± SD (cm)	176.3 (10.2)	172.0 (8.9)	172.5 (9.9)
Weight mean ± SD (kg)	70.2 (13.1)	78.3 (18.1)	75.1 (13.7)
Handedness (*N*_right_/*N*_left_)	17/3	20/2	20/1
History of upper limbs problems (N)	2	7	5

Detailed information about the age and sex group memberships for each individual IDs of the participants (participantIDs) can be found in the Supplementary Material Table S3.

All participants reported as healthy with normal to corrected-to-normal vision and hearing, as well as normal tactile sensation. Participants were cognitively intact, asymptomatic adults without occupational or recreational exposure to highly repetitive or strenuous hand exertions (e.g., repeated forceful dynamic grasping or prolonged static holds). The individual history of hand, forearm, elbow, neck or shoulder (i.e., upper limbs) problems, if any, were reported and recorded. Furthermore, the Edinburgh inventory^34^ was used to determine each participant’s handedness score.

### Acquisition setup

#### Stimulus videos

The stimulus videos of the hand movements were recorded using a Sony ILCE-6500 Camera with a frame rate of 29.97 frames per second (fps) and a spatial resolution of 3840 × 2160 pixels. The camera was positioned on a tripod approximately 1.5 m away from the performer. All videos were recorded under the same artificial light conditions in front of a white background. The performer wore neutral clothes and performed all movements with the right hand. Only the performer’s arm and hand were recorded. The recorded movements belonged to three categories adapted from the Ninapro database^13^ and described in the Introduction (i.e., A: basic finger movements, B: hand postures and wrist movements, C: grasps and functional movements; see Fig. 1 for their actionIDs and static images of recorded movements and Table S2 in Supplementary Material for detailed descriptions). In the postproduction phase the videos were edited with the software iMovie 10.1.14 and 10.3.5 (https://www.apple.com/imovie/). The videos were cut to a duration of 5 s for categories A and B, and 10 s for category C. The sound was removed from the video clips and a gray filter (preset grayscale template) was applied to the videos. Additionally, all videos were also flipped along the vertical axis to be used as stimuli for the left-handed participants. Thus, the instruction stimulus videos are available for showing the respective actions both with the right, as well as, with the left hand.

#### Anthropometric data

Hand anthropometry was recorded for all participants using a light-based 3D scanner (Artec EVA and Artec Studio version 15, www.artec3d.com). The Artec EVA is a portable handheld 3D scanner with the ability to scan 16 frames per second with ± 0.1 mm accuracy and 0.2 mm resolution. The position of the joints and other characteristic hand landmarks were recorded via a 3D camera image of the participants’ dominant hand with geometry tracker in a static flat pose with all fingers spread (flat spread hand; see Fig. 5). For each participant a final 3D-hand model was created with the automatic processing mode and saved in ‘‘.stl’’ format in Artec Studio Version 15. Furthermore, the models were registered with the 3-2-1 method, cropped and cleaned with Geomagic Wrap Version 2017.0.2.18 (https://oqton.com/geomagic-wrap/) and saved as preprocessed models with the extension _reg.stl. Additionally, key hand anthropometric measurements (i.e., hand length, width of hand and wrist) were taken manually with a generic measuring tape and digital caliper (stated accuracy ± 0.2 mm) following the anthropometric measurement template of the KIT Whole-Body Human Motion Database^35^. The hand length was indexed as the distance between the wrist and tip of middle finger. Wrist width was measured as the ulnar styloid, and hand width as the width at the first knuckles (metacarpophalangeal joints).

#### Kinematic data

Kinematic hand movements were recorded for all participants using a 22-sensor CyberGlove III dataglove (CyberGlove Systems LLC, www.cyberglovesystems.com). According to the respective participant’s handedness, either a left or a right glove was used for data recording. Data from the CyberGlove were transmitted via a USB interface once every 100 ms (the highest rate the measurement system could support). Communication with the CyberGlove was implemented through gladius, which is a purpose-made application with a graphical user interface (GUI) written for x86-64 Debian-based systems using C++ 20^36^ with gtkmm and gstreamermm. The connection to the CyberGlove employed the libglove library version 0.6.2 (https://libglove.sourceforge.net). Due to the legacy nature of the libglove library, it was compiled on an Ubuntu Jaunty Jackalope 9.04 (2009) virtual machine using boost 1.39 and was linked to the main application (gladius) as a static library. During measurements, the state of the glove was polled using the local serial interface of the libglove library (LocalCyberGlove) on a separate thread in order to achieve continuous data retrieval. On the client’s side, each sensor state was represented in double precision floating point format in degrees. Generally, on the hardware side CyberGlove acquires the sensor readings as an integer number in the range of 0–255 via an analog-to-digital conversion process based on the output voltage of each individual sensor, and then converts the values into angles, expressed in radians^37^.

In order to facilitate the comparability of measurements taken from different individuals, a mandatory calibration procedure was performed before data collection for each participant. During the calibration, each participant was prompted to hold a specific gesture for 100 samples (i.e., the palm was placed on a flat surface, with fingers extended and the thumb perpendicular to the the rest of the fingers, see Fig. 4), after which the respective average value for each of the 22 sensors was calculated and used as a base (e.g., origin or 0.0). After successful calibration, subsequent measurements used this base as an offset for the retrieved readouts for the respective sensor. To ensure consistency, each participant was only allowed to complete calibration if the hand was kept motionless. In other words, if during the 100 sample assessments a sensor value exceeded a difference of over 25 degrees, the calibration was restarted. In addition to the initial calibration prior to data acquisition, a well-established post-processing calibration method was employed to re-calibrate the data (see the section below).

#### Post-processing re-calibration of kinematic data

Although our calibration process, performed prior to data collection, is designed to be brief and simple, it is important to acknowledge that it tends to yield results with limited precision (see Supplementary Material Figs. S1–S5). Ideally, a comprehensive and participant-specific calibration procedure that incorporates both gain and offset adjustments should be employed. The offset parameter influences the starting position for each joint’s range, representing the baseline values of the sensor output at resting position. Whereas, the gain parameter determines the extent of the permissible range of motion for each joint, considering anatomical constraints and individual hand anatomy. However, the proprietary CyberGlove software or established and verified protocols^38^ for such detailed calibration entails a complex and cumbersome process, rendering it unsuitable for lengthy data collection protocols or general users. To address this challenge, we implemented a post-processing calibration method using the open-source data and protocol provided by Gracia-Ibáñez and colleagues^38,39^. Specifically, the mean gain values for each sensor, as well as specific cross-coupling effect corrections were derived from the BE-UJI code^39^ and utilized as a sensor data correction technique to generate coherent and realistic data. By adopting this alternative calibration method, we aim to mitigate the limitations of our brief calibration process and enhance the reliability and validity of the obtained data (see also^17,40,41^). Within the *CeTI-Age-Kinematic-Hand* database^42^, both the only offset-calibrated and re-calibrated data are published and available for users (see Fig. 3). This approach allows for greater accessibility and ease of use, particularly in the context of extensive data collection protocols or for researchers who may want to investigate alternative post-processing approaches.

### Acquisition procedure

Participants sat at a desk in a chair, adjusted for maximum comfort, while resting their arms on the armrests. A PC screen (ASUS VG248QE, 24 inch, max. refresh rate 144 Hz), connected to the acquisition laptop in front of the participant, displayed visual stimuli (instruction text and videos) for each movement exercise, while also recording data from the CyberGlove (i.e., the kinematic data acquisition device) of the participant’s dominant hand. Before the data recording started, participants were familiarized with the CyberGlove and given a general overview of the types of movements they had to perform. They were informed about how the movements and anthropometry of their dominant hand would be recorded.

A recording session with the CyberGloves started with a start panel on the GUI where participant-specific ID (participantID), session-related (session ID), CyberGlove handedness, file directory, demographic (age, sex), and anthropometric (handedness) information were recorded and the CyberGlove was calibrated. A calibration consisted of participants placing their gloved hand flat on the desk surface and spreading their thumb perpendicular (see Fig. 4). After completing the calibration procedure, participants went through a training session. During training, all stimulus videos were shown to the participant in the same fashion as they were subsequently shown during the experimental session. No kinematic data was recorded during training, but the participants were encouraged to familiarize themselves with the movements and the stimulus videos. No time restrictions were given during training. In case of uncertainty, an experimenter was in the room with the participant to answer any questions.

Next, the participants were guided through the experimental session block by block. Within a block, one of the three hand movement types (i.e., A: basic finger movements, B: hand postures and wrist movements, C: grasps and functional movements) was presented. That is, each block contained all movements of a given category and the movements were performed sequentially across trials in a predefined trial order (see Fig. 1). Each block contained two repetitions of the movements and three repetitions of each block were recorded, thus resulting in a total of 6 repetitions of each movement. Such movement repetition data are a basis for understanding age-related and individual differences in intra-individual variability. Between blocks the participants were allowed short rest periods to avoid muscular and cognitive fatigue.

**Fig. 2:**
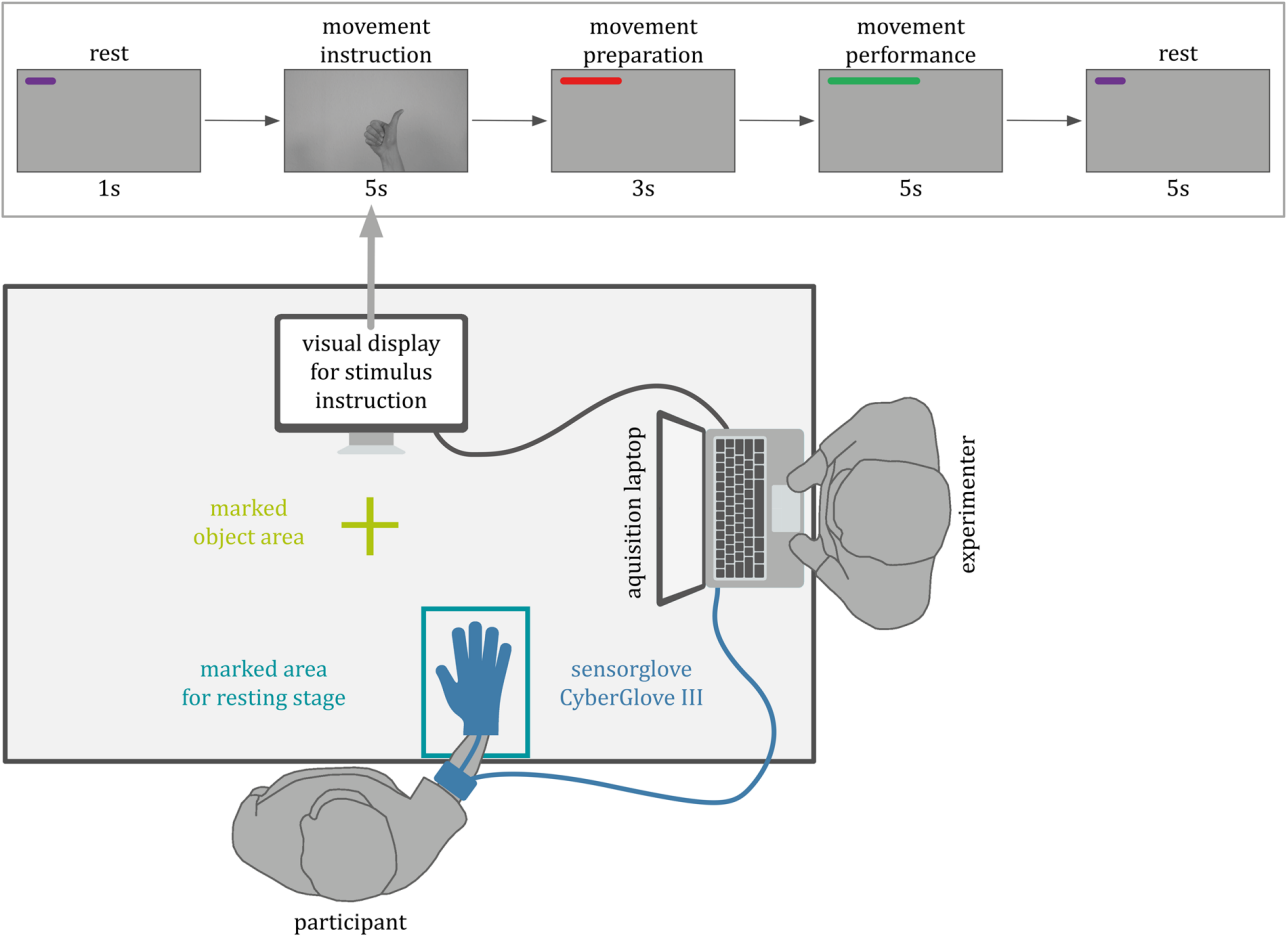
Schematic representation of the experimental setup for movement recording. A PC screen, connected to the data acquisition laptop, displayed the GUI, which guided the participant through the experiment (shown here is the setup for recording from a right-handed participant). Top inset: An instructional video was shown in each trial for the respective movement to be performed. After viewing the instruction video, the participants had to execute the movement at their own individual naturalistic movement speed during the movement performance period while wearing a CyberGlove on their dominant hand (see descriptions in the text for other details).

The top inset in Fig. 2 depicts the structure of a trial. Within each trial, participants were asked to mimic the movement that was shown in the short instruction video clip of that given trial. The temporal structure of a trial were indicated to the participants on the computer screen by means of written instructions and a timer accompanied with a color-coded horizontal bar that expanded horizontally on the top of the screen (see top inset Fig. 2). Each trial started with a 1 s rest period (pre-movement rest), followed by the movement instruction video for 5 s for movements of categories A and B (10 s for trials in category C). After viewing the instruction video, a countdown of 3 s with a dynamic horizontal bar displayed in the upper center of the screen signaled the participants to get ready for movement execution. This was followed by a screen with the horizontal bar turning green, indicating the start of movement execution (performance); the green horizontal bar expanded gradually to indicate the recording duration. After performing the movement, there was a post-movement rest period of 5 s. Importantly, during the period of movement performance, no stimulus video was shown in order to allow each participant to execute the movement naturally with an individual pace.

In order to ensure consistency, during the experiment the participants were instructed to place their gloved hand at a designated resting position marked on the table (see Fig. 2). For movements involving interactions with objects, another designated location at approximately 20 cm in front of the hand-resting position was marked as the object area. In a given trial involving grasping or functional movement, the participants were asked to lift the given object smoothly to a height of approximately 5 cm above the desk surface and to keep their grasp and lifting movement consistent throughout the process. The setups and procedure were done to ensure measurement consistency and quality across trials and participants. For a detailed description of all recorded movements see also Table S2 in the Supplementary Material.

## Data Records

**Fig. 3:**
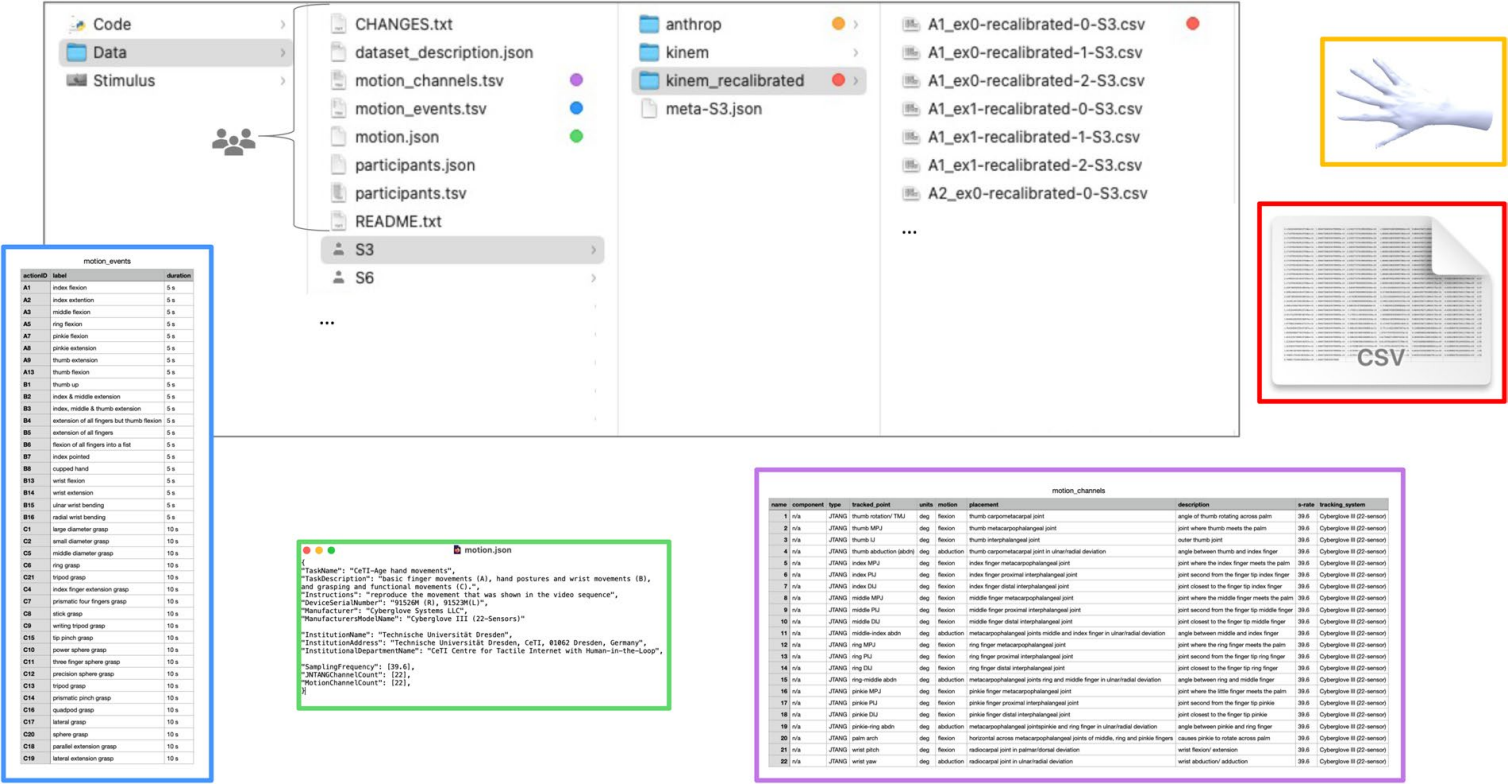
Folder structure of the *CeTI-Age-Kinematic-Hand* database. The left-most column shows the content of the entire database at the highest level. The 2nd column represents the highest level of the data folder structure, the 3rd column represents the level of an individual participant, and the right-most column represents the level of a specific participant’s re-calibrated kinematic data. Color-coded insets show selected motion meta files and sample 3D and kinematic data. The annotated screenshot provides an overview of the organizational hierarchy in the database.

The database adheres to the BIDS^43^ standard while incorporating our own extensions to accommodate kinematic and anthropometric data. This includes raw and re-calibrated kinematic data (“sensor data”) as well as anthropometric data (“3D data”) and is available in the figshare repository: *CeTI-Age-Kinematic-Hand*^42^. Following the BIDS convention, the data records have a folder structure as shown in Fig. 3, which begins with one subfolder for each of the 63 participants named with the specific ID (participantID), e.g., S3, S6, S7 etc. and various metafiles.

For instance, the participants.tsv file gives a detailed tabular overview of the general participant sample (participantID, age group, sex, handedness-glove, etc.), which are, in turn, defined in the participants.json file. The files dataset_description.json and README.txt provide an overview of all relevant database information and instructions. Additionally, the file CHANGES.txt contains the database history.

Inside each of the participant subfolders there are three further subfolders: kinem (for raw kinematic data), kinem_recalibrated (for adjusted kinematic data) and anthrop (for anthropometric data). Additionally, for each database entry (participant) there is a meta file (e.g., meta-S3.json) that contains the participant-specific information collected by the GUI and via REDCap (i.e.,demographic and anthropometric data).

Finally, the stimulus video set (“stimulus material”) is also provided in a folder called Stimulus, and custom code of the Technical Validation in a folder called Code.

### Sensor data

Table 2 provides an illustrative example showcasing how the naming convention of the kinematic data files contains information about the performed hand movement (i.e., actionID in categories A,B,C as shown in Fig. 1), a running counter ID (trialID labeled as “ex”, followed by the numerical value of trialID), the data type (offset-calibrated or “recalibrated”), the numerical value of the block and the participantID (labeled as “S” and followed by the numerical value of the specific ID). It is important to note that indexing for trialID and block starts at 0, which means that the first element in these sequences has an index of 0, the second element has an index of 1, and so on. For example, for the first entry in Table 2 the file name A1_ex0-0-S3.csv (see also Fig. 3) signifies that it contains offset-calibrated data derived from the initial trial (‘ex0’) of the first block (‘0’) for action A1 (‘A1’), performed by participant S3 with the corresponding ID (‘S3’). All files follow this naming scheme, where the labels are replaced by the corresponding values for actionID, trialID, (datatype; in case of re-calibrated data), block, and participantID.

**Table 2:** Naming convention of sensor data for an exemplary instance.

file name	actionID	trialID	data type	block	participantID
*A1_ex0-0-S3.csv*	A1	ex0	calibrated (offset)	0	S3
*A1_ex0-recalibrated-0-S3.csv*	A1	ex0	re-calibrated (post-processing)	0	S3

This table presents an exemplary instance demonstrating the naming convention utilized for the kinematic data and its associated labels. Specifically, it showcases the naming scheme for the first trial of the first block of action A1 performed by participant S3. The example highlights two distinct files that contain identical kinematic data, calibrated with either offset only (1^st^ entry) or with the described post-processing calibration method (2^nd^ entry).

**Fig. 4:**
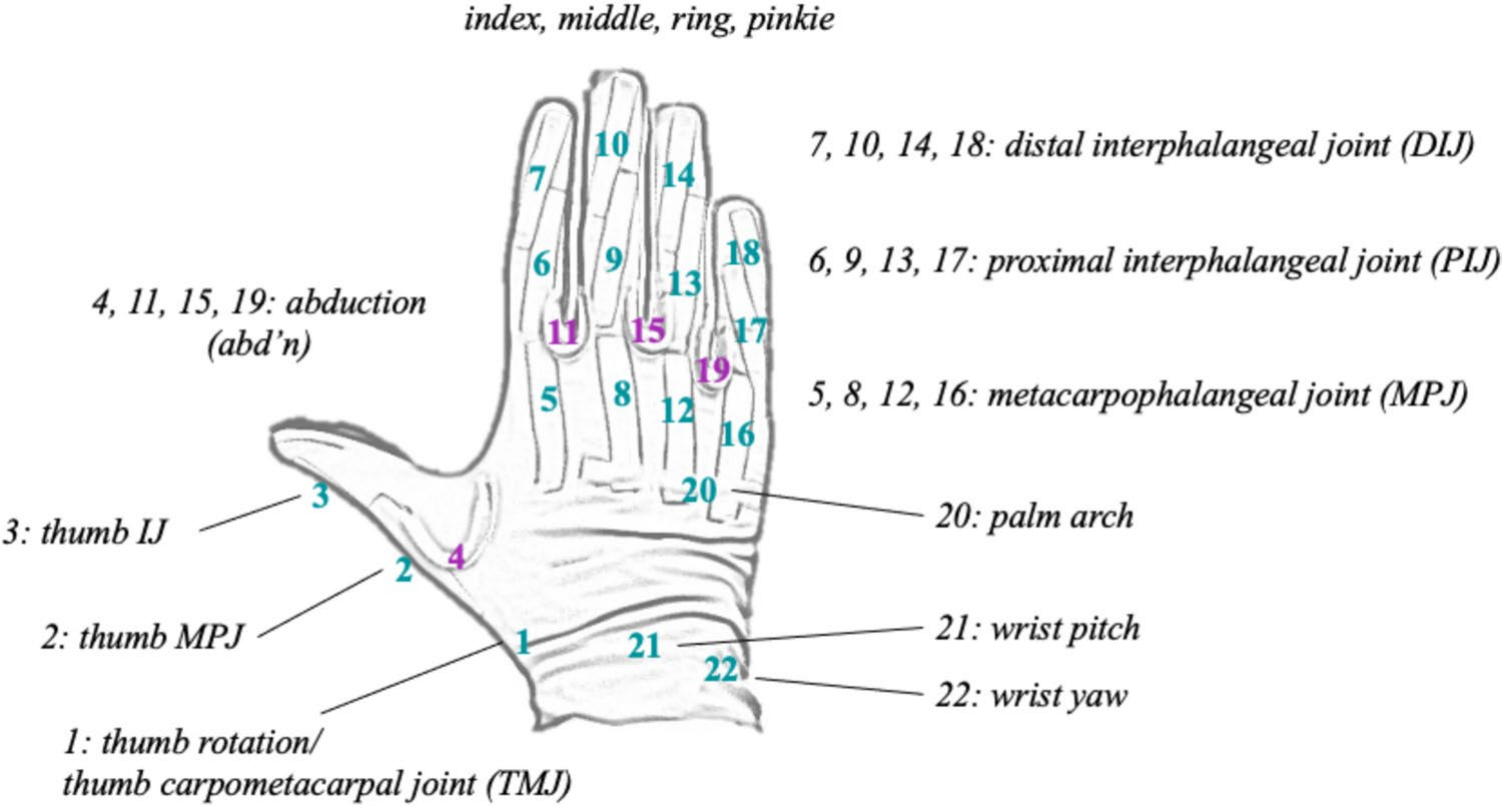
Resting (right) hand position with superimposed CyberGlove III sensorID labels. At the end and start of each trial participants assumed resting position, where their hand was placed flat on the table, fingers composed together and thumb perpendicular to the palm. This is also the specific gesture used for the calibration procedure. The placement of the sensor labels corresponding to the 22 columns (1–22; sensorID; abduction sensors in purple & bending sensors in teal) in the sensor data is superimposed.

Within a given “*.csv” action motion file, the rows represent samples recorded in frames (39.6 fps) during the movement performance time period (see Fig. 2 top inset “movement performance”). Timestamps were recorded in total elapsed milliseconds with the date of the data acquisition. Columns represent the CyberGlove sensorIDs 1–22 as depicted in Fig. 4. A detailed listing of the motion recording sensors and sensor descriptions can be found in the metafile motion_channels.tsv (see purple inset in Fig. 3) and Table S1 in the Supplementary Material.

### 3D Data

**Fig. 5:**
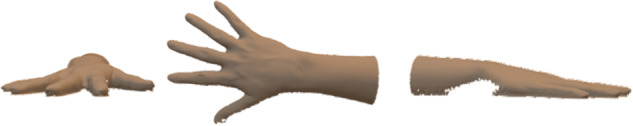
Preprocessed 3D hand model in distal, dorsal and ulnar view on the example of participantID S3.

For each participant, there is a anthropometric database entry that contains a preprocessed 3D model (in the form of “*.stl” files) of the participant’s dominant hand (i.e., the hand that was used to record the kinematic data with a CyberGlove). For example S3_reg.stl is the preprocessed 3D model based on the scanned individual hand anthropometric data corresponding to participantID S3 depicted in Fig. 5.

### Stimulus material

Lastly, the stimulus videos used for instructing all 40 hand movements for the right and left hand are also included in the database. Each video file is named according to the respective actionID (see Fig. 1 and Table S2 in Supplementary Material) and handedness (R-right, L-left). For instance, the file name, A1_R.mp4, labels the stimulus video corresponding to the execution of the hand movement with the actionID A1, flexion of the index finger, using the right hand.

## Technical Validations

As technical validations of the *CeTI-Age-Kinematic-Hand* database, we examined the recorded re-calibrated kinematic data by several key experimental conditions and used machine-learning to classify the recorded hand movements. Additionally, we have included the respective plots for the unadjusted offset-calibrated data in the Supplementary Material.

### Hand kinematics by experimental factors and individual differences

**Fig. 6:**
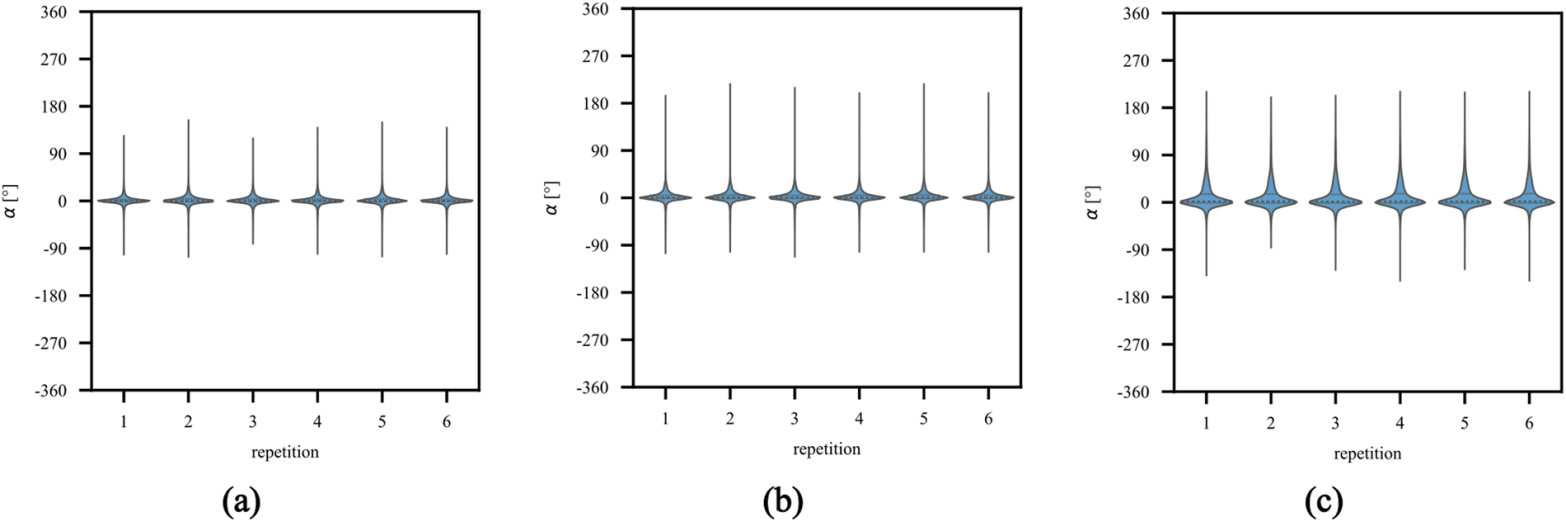
Violin plots displaying the angle distribution of the recorded kinematic data across all 22 sensors and participants over repetitions for the three different movement categories–basic finger movements (**a**), hand postures and wrist movements (**b**), and grasping and functional movements (**c**). The short dashed lines represent the 1st and 3rd quartiles, where as the long-dashed line indicate the median of the distributions.

**Fig. 7:**
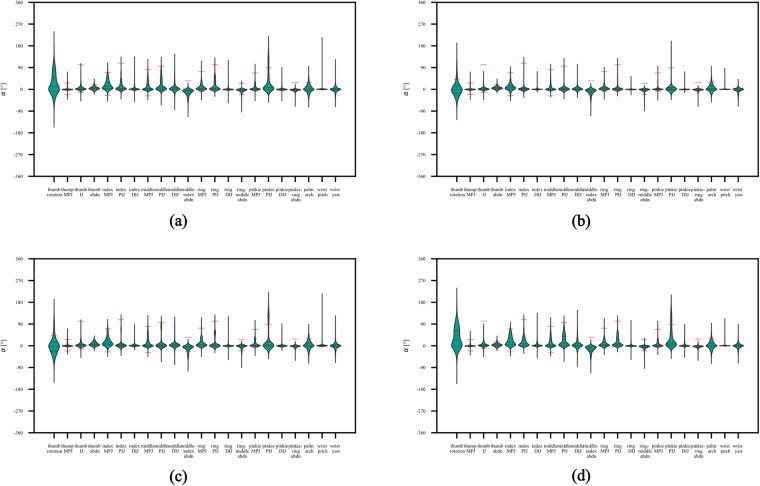
Violin plots displaying the joint angle distributions separately for each of the 22 sensor: aggregated data across all hand movements (**a**), basic finger movements in category A (**b**), hand postures and wrist movements in category B (**c**), and grasping and functional movements in category C (**d**). The horizontal short and long dashed lines represent the quartiles and median positions of the distributions. Global active range of motion (AROMs) reported in prior research^48^ are marked with red lines. Unmarked AROMs were not previously published.

**Fig. 8:**
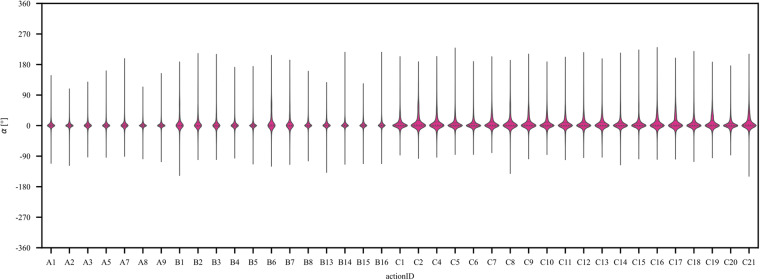
Violin plots of the mean joint angle distribution for each actionID. The inner horizontal lines represent the quartiles of the angle distribution. The center dashed line represents the median angle of among joints.

**Fig. 9:**
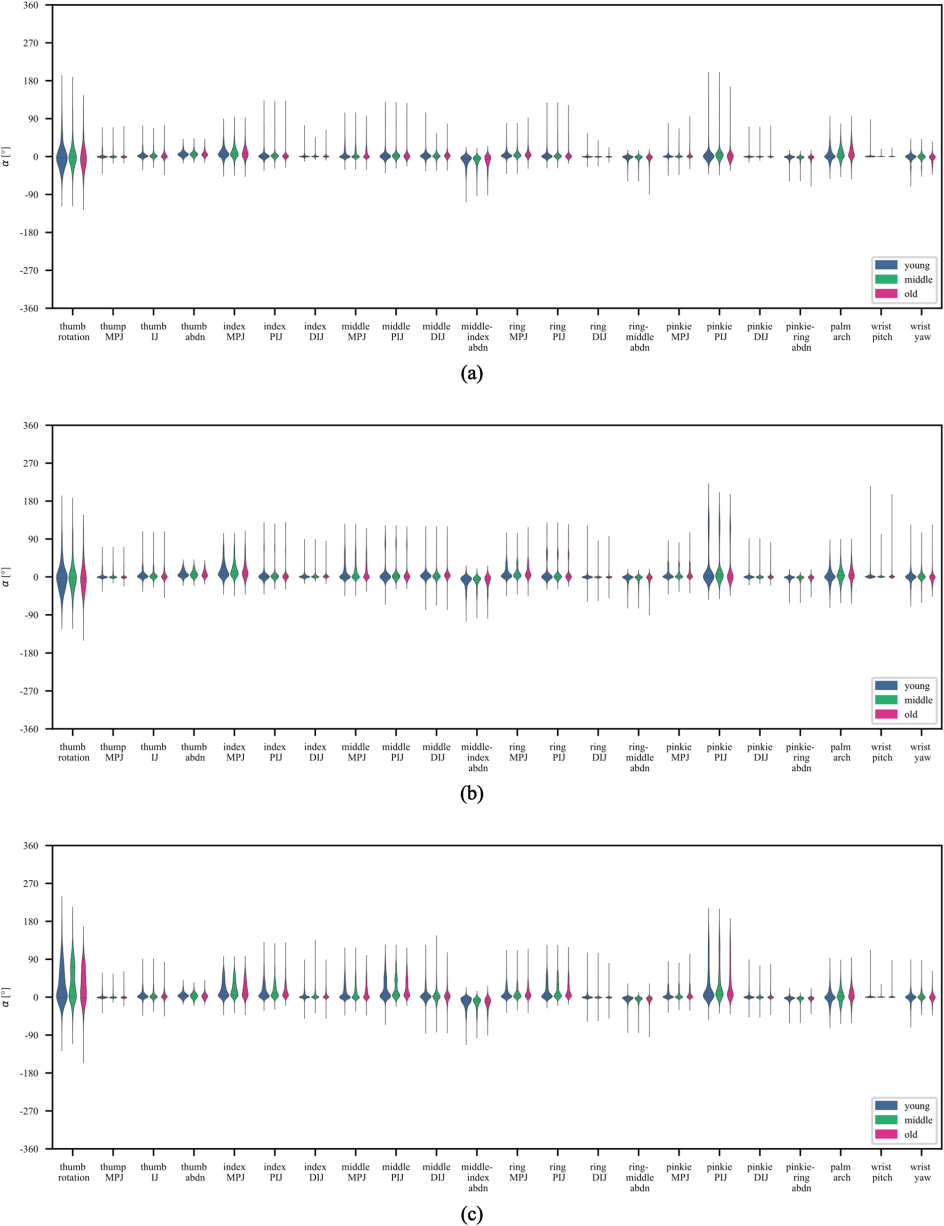
Violin plots depicting joint angle distributions over 22 sensors for the 3 movement categories, split by age group. Quartile Positions and Median Angles of exercises are shown with dashed lines.

This section shows results of descriptive analyses of the recorded data by experimental conditions to ensure quality control of the database. For this purpose, hand movement actions, age groups of the participants, and the range of joint angles assessed with the dataglove as an estimate of hand kinematics (see for example^17,44^) were employed to validate that the recorded kinematic data do vary with respect to experimental factors and yield individual and age differences. Furthermore, fatigue or adaptation to the movements^45^ may reflect in natural trial-by-trial fluctuations of the recorded movement data; thus, we also examined the data with respect to trial repetitions. To this end, we used Python^46^ 3.8.3 and seaborn 0.12.2^47^ to derive violin plots with a set kernel bandwidth of 0.2. The results are visually displayed as violin plots in Figs. 6–9, with values of the median and the quartiles (1st and 3rd) shown as long and short dashed lines, respectively.

#### Joint angle distributions of kinematic data over movement repetitions

First, we validated the recorded data across all participants and sensors with respect to movement repetitions separately for each of the three action categories. Based on previous validations of the Ninapro database^17,44^, we expected no substantial differences in the distributions of joint angles acquired by the CyberGlove over six movement repetitions. Our acquisition protocol was designed with the goal to minimize fatigue and movement adaptation, while also obtaining a sensible number of overall movement repetitions. Of note, differing from previous work^13^ which recorded hand movements performed simultaneously while participants viewed the stimulus videos, participants in the present database only executed the movements after seeing the respective movement instruction videos. Thus, the movement executions in our *CeTI-Age-Kinematic-Hand* database were of a more individualized and naturalistic nature. A setup of this kind can increase the generalizability of the recorded data and provide a broader variety of movement patterns for developing hand models or training machine learning (ML) algorithms to classify hand movements. The results can be seen in the three panels of Fig. 6, with each movement category exhibits a distinct distribution shape that is maintained over all six repetitions. Overall, the range of motion appears to be wide, showing that participants were not limited in their movement, and consistent across all repetitions, indicating that participants showed little change of intrinsic characteristics over time (e.g., adaptation, fatigue) at the group level.

#### Joint angle distributions of kinematic data over sensors by movements

Second, we next validated the data at the level of individual sensors by movements. Given the wide selection of hand movements (see Fig. 1), we expected that the joint angle distributions of the recorded kinematic data would differ between movement. By design the database includes different movement categories, which itself involve the use of different muscles, different fingers, and manipulations of different objects (see also grasp taxonomies e.g.,^29,30^).

The distributions of joint angles averaged across all movement categories and participants at the sensor level are displayed in Fig. 7a. It shows that different joint angles recorded by the 22 sensors varied substantially during the recorded exercises. As the CyberGlove was calibrated and the neutral postures was maintained, both at the start and the end of each movement execution for each participant, the depicted angle distributions are zero-centred. In addition, Fig. 7b–d show the same validation by separately for the three different movement type categories (A, B, and C). These plots show the variability and average movement angles of the joints corresponding to their respective sensors for all movements in the same category. Of note, one can see, for example, a transition in the different thumb joint angle distributions as the hand movements change from individual finger flexion exercises (see Fig. 7b) to grasp-centered exercises (see Fig. 7d). Moreover, in the figures depicting individual joint angles (Fig. 7), red horizontal lines are overlaid to represent the average active range of motion (AROMs) previously documented in the literature^48^. Previous studies have demonstrated that these ranges closely align with functional ranges of movement, with a reported deviation of up to 28° during activities of daily living^41,48^. As evident from these figures, the majority of our recorded and re-calibrated joint angles fall well within the established range of these values.

More specifically, in Fig. 8 the distributions of joint angles at the level of the 40 individual hand movements (i.e., actionIDs) are shown. While for basic finger movements (category A), the join angles are mostly centered around 0°, the more complex movements, (i.e. hand postures and wrist movements or functional and grasping movements; categories B and C) show an increasingly scattered distribution of joint angles. This is in line with our expectation, that these movements are more complex and involve the usage of the whole hand, and therefore show greater average angle variability across sensors and participants.

#### Joint angle distributions of kinematic data over sensors by age groups

Third, given age-related differences in movement variability had been reported in previous studies^20,21,23^, we examined the variability of hand kinematics at the sensor level by the age groups of the participants. In Fig. 9 the joint angle distributions for movements within each movement category is presented separately for the 22 sensors corresponding to specific joints in the hand and for each of the three age groups. Whereas the general trends are comparable, the plots also reveal variabilities between age groups.

**Fig. 10:**
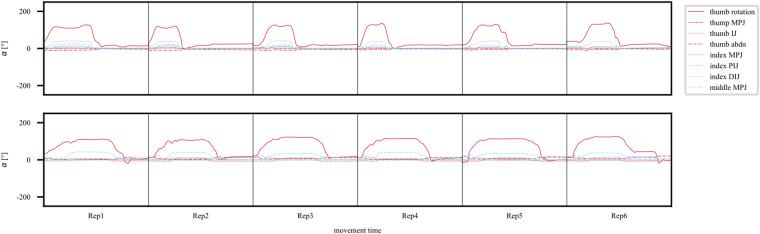
Exemplary concatenated angle trajectories for a large diameter grasp movement (actionID C1). The data shown here were recorded from the thumb and index finger sensors (see legend for details). In the recordings one young participant (top panel) and one old participant (bottom panel) performed all six movement repetitions (Rep; separated by vertical lines).

Furthermore, in Fig. 10 one can see exemplary data for one grasp movement of two participants of different ages across movement repetitions. One young (S17) and one old (S23) participant each reached towards and picked up an object (1.5 l standard PET water bottle, 8.6 cm diameter) with the large diameter grasp, lifted it about 5 cm off the table, and put it back on the table. The concatenated raw angle trajectories show all six respective repetitions of the movement. One can see that the data of the old participant (bottom panel) exhibited greater movement variability and longer movement durations.

### Movement classification

**Fig. 11:**
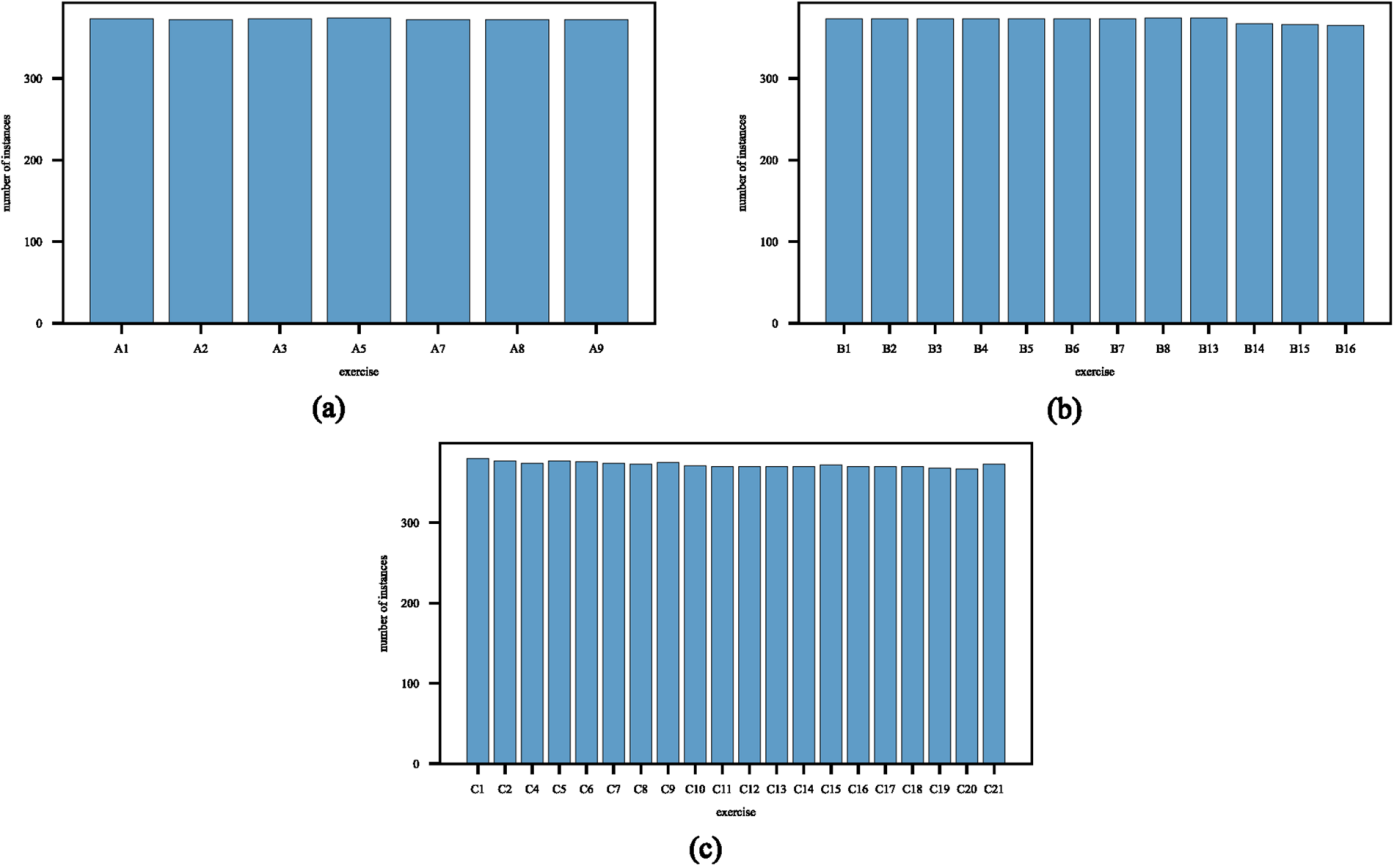
Number of available instances for each hand-movement exercise (actionID).

To test whether the recorded data is of high quality allowing movement classifications, the multivariate time series of the kinematic data obtained from the CyberGlove sensors were analysed. Specifically, the objective was to determine whether the different movements within a category could be correctly identified in terms of specific hand movements (i.e., by actionID). The selection of feature extraction and classification algorithms was based on prior wearable sensor ML work^44,49–51^. The feature extraction involved the computation of sensor-wise root-mean-square (RMS), empirical variance (var), and mean time series values. These features were calculated for each of the 22 sensors and combined to form a comprehensive set of features representing the entire hand motion. The discrimination of movements within each category was performed using four machine learning algorithms: Random Forest^52^, 5-nearest-neighbor classifier (KNN)^53^, linear-discriminant-analysis (LDA)^54^ and a support-vector-classifier (SVC)^55^. All classifiers were implemented using Python^46^ 3.8.3 and Scikit-learn^56^ 1.0.2 with corresponding default settings, and verified using a 20-fold cross-validation. Classification accuracy and F1 score^57^ were utilized as performance metrics and are shown in Table 3. In balanced datasets, containing an equal number of instances in each classification category, accuracy is a common measure of classification performance. However, in the case of imbalanced datasets, F1-score is often used^57^. Our dataset is slightly imbalanced (see Fig. 11 and supplementary Table S3 due to some missing data (kinematic sensor data) resulting from occasional participants’ erroneous movement executions or due to technical issues during the recording. As such, to adequately evaluate classification performance, both accuracy and F1-score are reported.

**Table 3:** Performance evaluation of four employed classifiers in classifying movement categories.

	Category A	Category B	Category C
*KNN*_*accuracy*_	0.907 (0.028)	0.775 (0.025)	0.744 (0.028)
*LDA*_*accuracy*_	0.914 (0.020)	0.760 (0.024)	0.659 (0.026)
*RF*_*accuracy*_	**0.944 (0.021)**	**0.844 (0.026)**	**0.793 (0.022)**
*SVC*_*accuracy*_	0.938 (0.023)	0.757 (0.025)	0.757 (0.023)
*KNN*_*F1*_	0.908 (0.027)	0.772 (0.026)	0.745 (0.028)
*LDA*_*F1*_	0.915 (0.019)	0.759 (0.025)	0.659 (0.026)
*RF*_*F1*_	**0.944 (0.021)**	**0.841 (0.026)**	**0.792 (0.023)**
*SVC*_*F1*_	0.938 (0.023)	0.798 (0.026)	0.758 (0.023)

The table presents the mean accuracy and F1 scores used to evaluate the performance of the four employed classifiers: K-Nearest Neighbors (KNN), Linear Discriminant Analysis (LDA), Random Forest (RF) and Support Vector Classifier (SVC). The classification task involves categorizing movements into three categories: A, B, and C. The values provided in the table represent the mean scores, accompanied by their corresponding standard deviations in in parentheses. Bold values indicate the highest scores within that category.

The slight imbalance in the amount of available incidences per category in the dataset notwithstanding, the results presented in Table 3 indicate that the data of very good quality for movement classifications. The classifiers were successful in distinguishing between the different hand movements within a category, with mean accuracies between 65.6% and 94.4%, and mean F1-scores between 0.659 and 0.944. The Random Forest classifier demonstrated the best performance overall, achieving a mean accuracy score of 94.4% for category A, 84.4% for category B and 79.3% for category C. In general, category A, which had relatively fewer movements and involved only one finger or joint (that went to one of the two most extreme positions, e.g., flexion or extension), showed the most distinguishable kinesthetic data pattern and yielded the highest performance. In this movement category all classifiers achieved nearly identical results between each other and in terms of accuracy (ranging from 90.7% to 94.4%) and F1-scores (range: 0.908–0.944). For the movements in category B, the classifiers achieved a mean accuracy ranging between 75.7% and 84.4%, and a mean F1-score between 0.757 and 0.841. Finally, the applied classifiers were able to classify the actionIDs with accuracies between 65.9% and 79.3% and F1-scores between 0.659 and 0.792 for category C. This is as expected, because the grasp and functional movements were much more complex and consisted of, at least partially, similar grasp movements and object interactions.

**Fig. 12:**
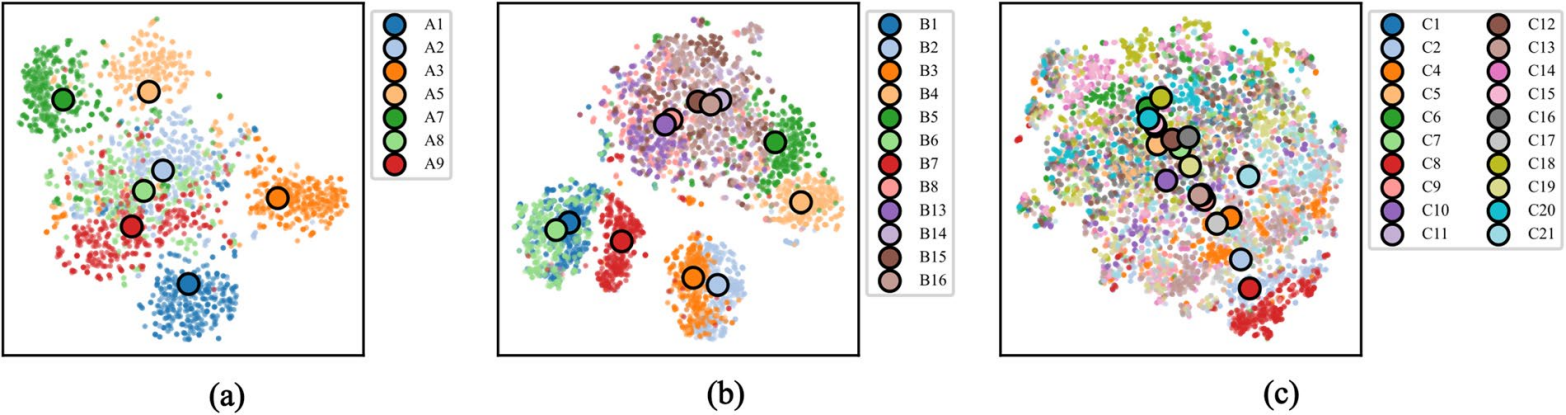
t-SNE embeddings of sensor data employed on the three different exercise categories. Scatters represent individual embeddings and larger circles indicate actionID centroids.

In order to visualize the discriminability of the kinematic data records, a 2-dimensional embedding was generated using t-distributed stochastic neighbor embedding (t-SNE)^58^. To this end, the sensor-wise data of each hand movement recording was concatenated into a single vector. Thus, the information of all sensors were used in this dimensionality reduction. The t-SNE embedding implementation of Scikit-learn^56^ 1.0.2 was utilized with the perplexity set to 50. The resulting 2-dimensional vectors along with the centroids of the embedded data instances that belong to the same actionID are visualized in Fig. 12.

The t-SNE results were found to preserve much of the global geometry observed in the classification results (see Table 3). This provides a visualization of the high-dimensional kinematic data that goes beyond the standard numerical performance metrics. Specifically, on the one hand, the t-SNE embedding produced more distinguishable embedded clusters of hand movements for category A and B (see Fig. 12a,b). On the other hand, the clusters of recordings of movement in category C are placed closer together, more overlapping, and conflated (see Fig. 12c), making them more difficult to distinguish from each other. The presented projection serves as a useful analytical tool; however, it is important to acknowledge its inherent limitations as they offer incomplete approximations of the full dataset, potentially resulting in the loss or obscuring of certain data aspects. While Fig. 12 illustrates the clustering of movements in a low-dimensional space, it provides limited insights into the underlying factors driving the clustering. As such, dimensionality reduction is employed only for technical validation purposes in this data descriptor. To ensure the reliability of conclusions drawn from the reduced data representations, such as clustering or the identification of kinematic movements, it is imperative to validate them against the original high-dimensional data.

## Usage Notes

This novel hand movement database (*CeTI-Age-Kinematic-Hand*) can be used for a wide range of different domains, starting from virtual reality to robotics, to healthcare. We provide valuable data for various different use cases and potential applications, for example ML-based use cases such as the classification of grasping or social gestures, or to recognize different types of hand actions. Researchers can also use the data to identify movement patterns and make predictions to enhance human-computer interaction, facilitate rehabilitation and prosthetics, improve security by developing methods for perturbing movement, and enable advanced TI-technologies for immersive, multimedia digital environments. Of note, our representative data (incl. chronological age, sex, and hand dominance) naturally offers various sources of variability, such as natural trial-by-trial variability of the performed movements, and inter-subject variability over a wide adult age range in three age cohorts, and thus also potential differences in individual movement experiences and fitness. Older adults, for example have unique limitations when it comes to hand kinematics. Thus, by including this age group in the development and implementation processes, we can ensure that the TI-technology is accessible, sustainable and usable for everyone.

Within the *CeTI-Age-Kinematic-Hand* database^42^, both the offset-calibrated and re-calibrated data based on previous work^38^ are published. This approach allows for greater accessibility and ease of use, particularly in the context of extensive data collection protocols or for researchers who may want to investigate alternative post-processing data calibration approaches^3^. The selected hand movements were adapted from the Ninapro Project^13^ and labeled using the same naming convention, and therefore provide the possibility of comparing or matching datasets. If datasets were to be combined, the differences in the data acquisition protocol (baseline hand posture, movements not mimicked in temporal synchrony with stimulus videos, instead more naturalistic individual movements at own pace) should be taken into consideration. Furthermore, the (*CeTI-Age-Kinematic-Hand*) database includes anthropometric data that can be used to improve classification accuracy and modeling. In this way, the data could be used for motion analysis and synthesis, as well as animations. Researchers should be aware of the limitation in the data due to the usage of the CyberGlove III. For example, the glove might lead to problematic results due to size-fit issues. In particular, the distal phalanges (DIP) sensors are likely to provide reliable output when a participant’s hand fits the glove properly. However, smaller hands could result in poorer fit and subsequently only partial results. Additionally, some object interactions were difficult due to the decreased tactile feedback. This is in line with findings that bare hands are more efficient than gloved hands^59^. Nonetheless, data gloves offer many benefits as whole-hand input devices because they are relatively natural input devices with a high ease of use, especially for gestures and interactions with 3D objects^3^. Another benefit is that they do not suffer from occlusion like camera-based systems, thus providing the participants with a realistic and safe environment resembling real-world hand movements, and researchers with the means to collect large-scale hand movement data.

### Supplementary information

Supplementary Information SDATA_23_00880A

## Data Availability

The preprocessing of the kinematic data (removal of unused columns in.csv files (see Table S1, checking for corrupt files, removal of sensitive data, naming and sorting of files into folders) as well as the normalization of the kinematic data was performed using custom Python^46^ code, and can be found in the folder called Code within the *CeTI-Age-Kinematic-Hand*^42^ database. The custom Python scripts^46,47,56^ used for the descriptive analyses and ML-based movement classifications reported in the Technical Validation section can also be found in the Code folder of *CeTI-Age-Kinematic-Hand*^42^.
