# Supplementary material for: Coming in handy: CeTI-Age — A comprehensive database of kinematic hand movements across the lifespan
Source: Sci Data. 2023 Nov 25;10:826. doi: 10.1038/s41597-023-02738-3 (PMC10676381; doi:10.1038/s41597-023-02738-3)
Supplement: Supplementary file 1 — Supplementary Information SDATA_23_00880A [file 41597_2023_2738_MOESM1_ESM.pdf]

Supplementary Information. Coming in handy: *CeTI-Age* — A comprehensive Database of kinematic Hand Movements across the Lifespan

Contents

1 Sensor information 2

2 Action description 3

3 Participants 5

4 Technical Validation plots prior to re-calibration 5

References 10

1 Sensor information

sensorID	Byte Index (Cyberglove)	Sensor Name	Description
1	0	thumb rotation/ TMJ	angle of thumb rotating across palm
2	1	thumb MPJ	joint where thumb meets the palm
3	2	thumb IJ	outer thumb joint
4	3	thumb abduction (abdn)	angle between thumb and index finger
5	4	index MPJ	joint where the index meets the palm
6	5	index PIJ	joint second from the finger tip
7	6	index DIJ	joint closest to the finger tip
	7*	index abdn (not used)	sideways motion of index finger
8	8	middle MPJ	
9	9	middle PIJ	
10	10	middle DIJ	
11	11	middle-index abdn	angle between middle and index finger
12	12	ring MPJ	
13	13	ring PIJ	
14	14	ring DIJ	
15	15	ring-middle abdn	angle between ring and middle finger
16	16	pinkie MPJ	
17	17	pinkie PIJ	
18	18	pinkie DIJ	
19	19	pinkie-ring abdn	angle between pinkie and ring finger
20	20	palm arch	causes pinkie to rotate across palm
21	21	wrist pitch	flexion/ extension
22	22	wrist yaw	abduction/ adduction
	23	(not used)	
	24	(not used)	

Table S1. Sensor data sensorID and byte ordering with sensor names and descriptions. SensorIDs correspond to the 22 columns (1-22) in the kinematic data of the database[1]. * according to the Cyberglove III manual[2]: "absolute abduction sensor not implemented. Refer to middle-index relative abduction sensor. In the raw data files Byte Index 7 and 23 return no values as they are not implemented by Cyberglove. Additionally Byte Index 24 is reserved in the implemented custom software and always set to 0.0. See also Fig. 4 of the main text for sensor locations on 22-sensor Cyberglove III. (SensorID, Sensor Name and Description of this list might be used as alternative text for Fig. 1 in the main text.)

2 Action description

actionID	Label	Description
A1	index flexion	Index finger is flexed in palmar direction in a 90° angle.
A2	index extention	Index finger is extended in dorsal direction.
A3	middle flexion	Middle finger is flexed in palmar direction in a 90° angle.
A5	ring flexion	Ring finger is extended in dorsal direction.
A7	pinkie flexion	Pinkie is flexed in palmar direction in a 90° angle.
A8	pinkie extension	Pinkie is extended in dorsal direction.
A9	thumb extension	Thumb is extended in dorsal direction.
A13	thumb flexion	Thumb is flexed in palmar direction in a 90° angle towards the base of the pinkie.
B1	thumb up	Thumb raised up, all other digits flexed into a fist.<br>Represents "ok" gesture, or 1 in European style finger-counting.
B2	index & middle extension;<br>ring, little finger & thumb flexion	Index and middle fingers extended and raised up, all other digits flexed into a fist.<br>Represents "peace" or "victory" gesture, or 2 in American style finger-counting.
B3	index, middle & thumb extension;<br>ring and little finger flexion	Thumb, index and middle finger extended and raised up, all other digits flexed into a fist.<br>Represents 3 in European style finger-counting.
B4	extension of all fingers;<br>thumb flexion	Thumb flexed into palm, all other fingers extended and raised up.<br>Represents 4 in American style finger-counting.
B5	extension of all fingers	All fingers extended, raised up and splayed.<br>Represents 5 in Western world style finger-counting.
B6	flexion of all fingers into a fist	All fingers flexed into a fist, with thumb on top.
B7	index pointed	Thumb and index finger extended and raised up.<br>Represents "finger gun" gesture, or 2 in European style finger-counting.
B8	cupped hand	All fingers extended, raised and closed together, palm arched to make hand into shape of a bowl.
B13	wrist flexion	All fingers extended, raised and closed together, wrist flexed (palmar).
B14	wrist extension	All fingers extended, raised and closed together, wrist extended (distal).
B15	ulnar wrist bending	All fingers extended, raised and closed together, wrist bend towards the ulna.
B16	radial wrist bending	All fingers extended, raised and closed together, wrist bend towards to the radius.
C1	large diameter grasp	Reaching out and picking up a 1.5 l standard PET water bottle (8.6 cm diameter)<br>with the large diameter grasp, lifting it about 5 cm off the table, and putting it back on the table.
C2	small diameter grasp	Reaching out and picking up a long cylindrical stick (15 mm diameter)<br>with the small diameter grasp, lifting it about 5 cm off the table, and putting it back on the table.
C5	middle diameter grasp	Reaching out and picking up a 0.5 l standard PET water bottle (6.1 cm diameter)<br>with the middle diameter grasp, lifting it about 5 cm off the table, and putting it back on the table.
C6	ring grasp	Reaching out and picking up a horizontal 0.5 l standard PET water bottle (6.1 cm diameter)<br>with the ring grasp between thumb and index finger, lifting it about 5 cm off the table, and putting it back on the table.
C21	tripod grasp	Reaching out and twisting off the cap (30 mm diameter) of a 0.5 l standard PET water bottle with the tripod grasp of thumb,<br>index and middle finger, lifting it about 5 cm off the bottle, and putting it back on the bottle.
C4	index finger extension grasp	Reaching out and picking up a plastic knife with the index finger extended on top,<br>lifting it about 5 cm off the table, and putting it back on the table.
C7	prismatic four fingers grasp	Reaching out and picking up a standard pen (7.5 mm diameter) with thumb opposite all other four fingers,<br>lifting it about 5 cm high out of the pen holder, and putting it back into the holder.
C8	stick grasp	Reaching out and picking up a standard pen (7.5 mm diameter) with only four fingers grasped around, while thumb extended,<br>lifting it about 5 cm high out of the pen holder, and putting it back into the holder.
C9	writing tripod grasp	Reaching out and picking up a standard pen (7.5 mm diameter) with three fingers in writing tripod grasp,<br>lifting it about 5 cm high out of the pen holder, and putting it back into the holder.
C15	tip pinch grasp	Reaching out and picking up a standard pen (7.5 mm diameter) with thumb and index finger in tip pinch grasp,<br>lifting it about 5 cm high out of the pen holder, and putting it back into the holder.
C10	power sphere grasp	Reaching out and picking up a standard tennis ball (66 mm diameter) with the five fingers and palm cupped around<br>in power sphere grasp, lifting it about 5 cm off the table, and putting it back on the table.
C11	three finger sphere grasp	Reaching out and picking up a standard tennis ball (66 mm diameter) with thumb, index and middle finger grasped around,<br>lifting it about 5 cm off the table, and putting it back on the table.
C12	precision sphere grasp	Reaching out and picking up a standard tennis ball (66 mm diameter) with the fingertips of all<br>fingers grasped around, lifting it about 5 cm off the table, and putting it back on the table.
C13	tripod grasp	Reaching out and picking up a standard tennis ball (66 mm diameter) with thumb, index and middle finger grasped around,<br>lifting it about 5 cm off the table, and putting it back on the table.
C14	prismatic pinch grasp	Reaching out and picking up a standard 2x2 LEGO brick with pinch grasp between thumb and index finger,<br>lifting it about 5 cm off the table, and putting it back on the table.
C16	quadpod grasp	Reaching out and picking up a standard 2x2 LEGO brick with grasp between thumb and index, middle and ring finger,<br>lifting it about 5 cm off the table, and putting it back on the table.
C17	lateral grasp	Reaching out and picking up a compact disk (CD) from the side with lateral grasp: The thumb pushes against<br>the side of the index finger to secure the CD, while lifting it about 5 cm off the table, and putting it back on the table.
C20	sphere grasp with disk	Reaching out and picking up a CD from the top with sphere grasp between thumb and all findertips,<br>lifting it about 5 cm off the table, and putting it back on the table.
C18	parallel extension grasp	Reaching out and picking up a book over its spine (15mm thick) with extended parallel grasp between thumb and palm<br>and fingers, lifting it about 5 cm off the table, and putting it back on the table.
C19	lateral extension grasp	Reaching out and picking up a plate (20.5 cm diameter) from the side with lateral grasp between thumb and flexed fingers,<br>lifting it about 5 cm off the table, and putting it back on the table.

Table S2. Detailed descriptive list of all actions with actionID, label and description. Actions are sorted by categories—basic finger movements(A), hand postures and wrist movements (B), and grasping and functional movements (C) and order of acquisition within each category. Visual presentations of each actionID can be found in Fig. 1. (This list might be used as alternative text for Fig. 1 in the main text.)

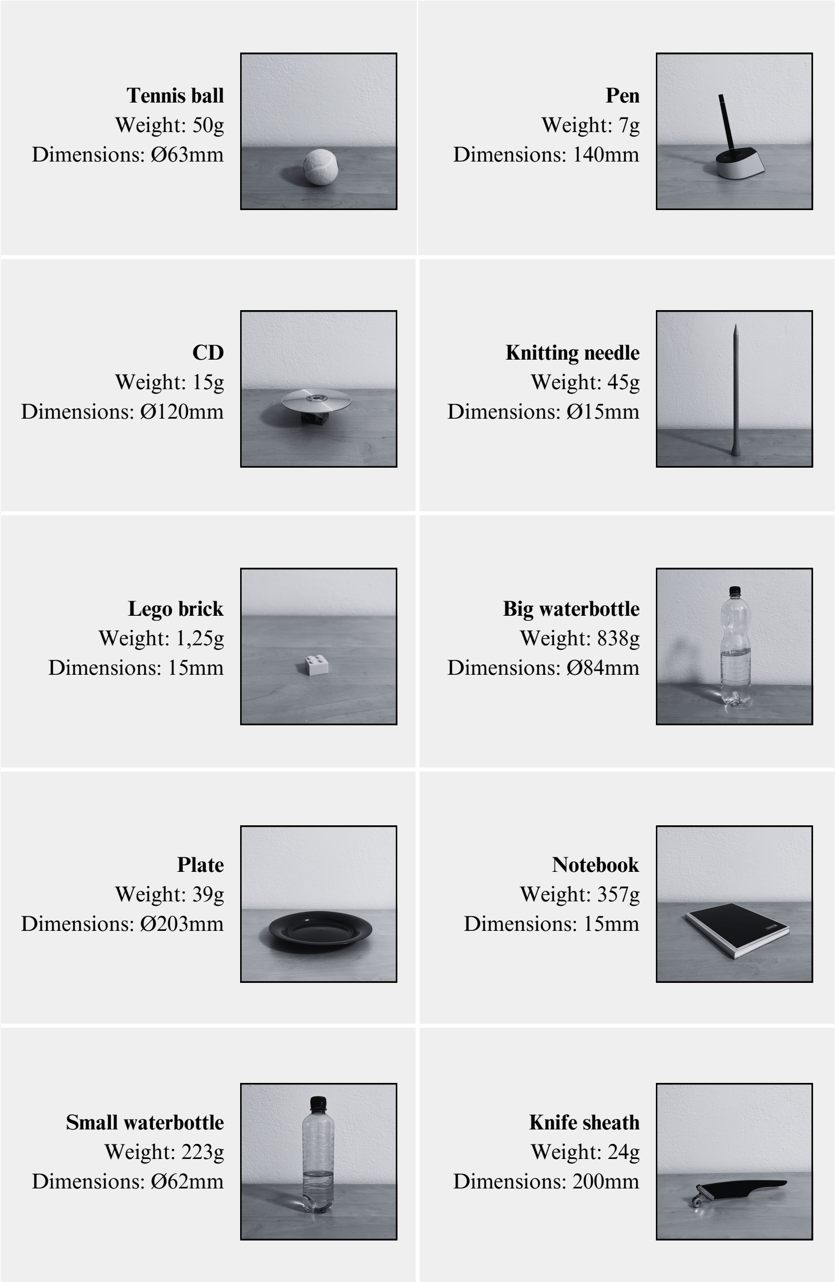

Figure S1. Objects used for grasping and other interactions in category C.

3 Participants

	Young Adults	Middle-aged Adults	Old Adults
**Female**	Su3 (6/6/6// 240)<br>Su6 (6/6/6// 240)<br>Su7 (6/6/6// 240)<br>Su13 (6/6/6// 240)<br>Su15 (6/6/6// 240)<br>Su42 (6/6/6// 240)<br>Su43 (6/6/6// 240)<br>Su46 (6/6/6// 240)<br>Su51 (6/6/6// 240)<br>Su72 (6/6/6// 240)	Su18 (4/4/5+// 190)<br>Su21 (6/6/5// 227)<br>Su22 (6/6/6// 240)<br>Su26 (6/6/6// 240)<br>Su27 (6/6/6// 240)<br>Su36 (6/6/6// 240)<br>Su47 (6/6/6// 240)<br>Su52 (6/6/6// 240)<br>Su53 (6/6/6// 240)<br>Su54 (6/6/6// 240)<br>Su57 (6/5+/6// 246)<br>Su61 (5/6/6// 223)<br>Su81 (6/6/6// 240)	Su29 (6/6/4+// 205)<br>Su32 (6/6/9// 282)<br>Su38 (6/6/6// 240)<br>Su40 (6/6/6// 240)<br>Su58 (6/6/6// 240)<br>Su62 (6/4+/6// 230)<br>Su63 (6/6/6// 240)<br>Su64 (6/6/6// 240)<br>Su65 (6/4+/5+// 233)<br>Su77 (6/6/6// 240)
**Male**	Su8 (6/6/6// 240)<br>Su9 (6/6/6// 240)<br>Su12 (6/6/4+// 211)<br>Su14 (4/6/6// 224)<br>Su16 (6/6/6// 240)<br>Su17 (6/6/6// 240)<br>Su25 (6/6/6// 240)<br>Su44 (6/6/6// 240)<br>Su71 (6/6/6// 240)<br>Su73 (6+/6/6// 242)	Su28 (6/6/6// 240)<br>Su34 (6/6/6// 240)<br>Su37 (6/6/5// 220)<br>Su45 (6/6/6+// 243)<br>Su48 (6/6/6// 240)<br>Su59 (6/6/6// 240)<br>Su75 (6/5+/5+/ 233)<br>Su78 (5+/6/6+/ 245)<br>Su80 (6/6/6/ 240)	Su11 (6/6/6// 240)<br>Su20 (6/5/6// 227)<br>Su23 (6/6/6// 240)<br>Su24 (6/6/4+// 210)<br>Su35 (6/5/6// 226)<br>Su49 (6/6/6// 240)<br>Su50 (6/6/6// 240)<br>Su55 (6/4+/6// 225)<br>Su56 (6/6/6+// 241)<br>Su67 (6/6/6// 240)<br>Su69 (6/6/6// 240)

Table S3. ParticipantID breakdown in age groups and sex. Values in brackets indicate for each participant the number of repetitions per block and total number of trials (A/B/C// N_{T}).

4 Technical Validation plots prior to re-calibration

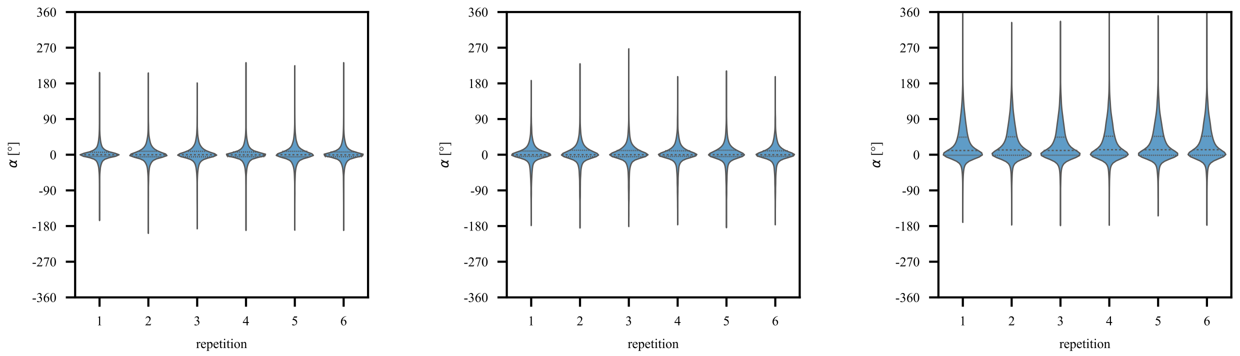

(a) Category A **(b)** Category B **(c)** Category C

Figure S2. Violin plots displaying the angle distribution of the recorded kinematic data that has only been offset-calibrated across all 22 sensors and participants over repetitions for the three different movement categories—basic finger movements (a), hand postures and wrist movements (b), and grasping and functional movements (c). The short dashed lines represent the 1st and 3rd quartiles, where as the long-dashed line indicate the median of the distributions.

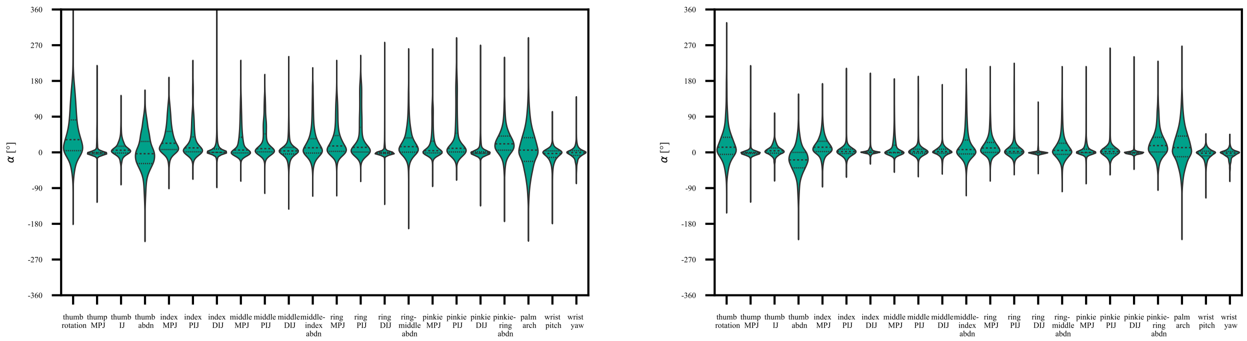

(a) Aggregated category A,B, and C **(b)** Category A

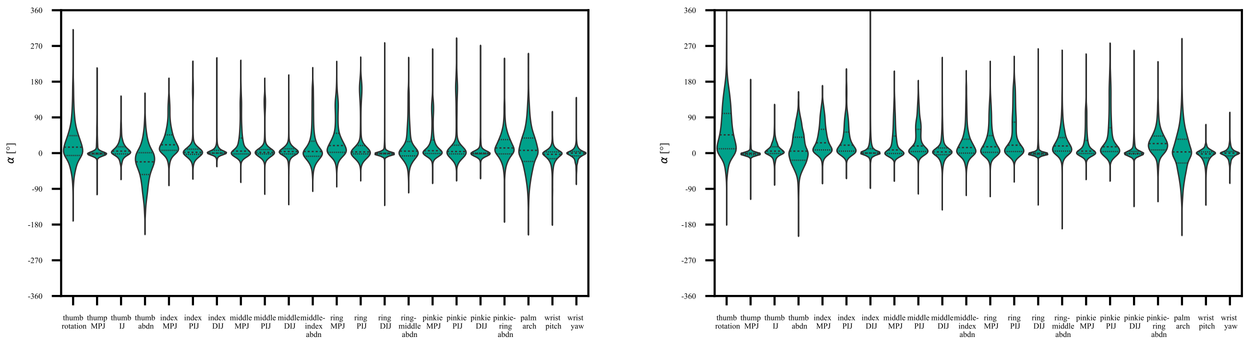

(c) Category B **(d)** Category C

Figure S3. Violin plots displaying the joint angle distributions before re-calibration separately for each of the 22 sensor: aggregated data across all hand movements (a), basic finger movements in category A (b), hand postures and wrist movements in category B (c), and grasping and functional movements in category C (d). The horizontal short and long dashed lines represent the quartiles and median positions of the distributions.

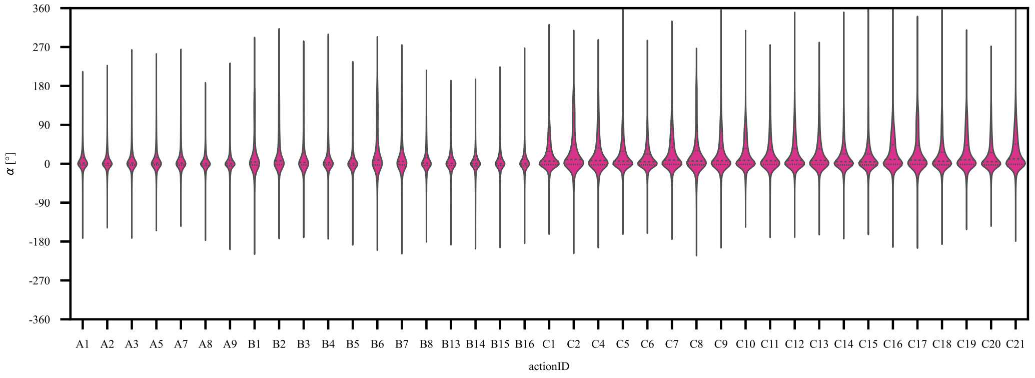

Figure S4. Violin plots of the raw/off-set calibrated mean joint angle distribution for each actionID. The inner horizontal lines represent the quartiles of the angle distribution. The center dashed line represents the median angle of among joints.

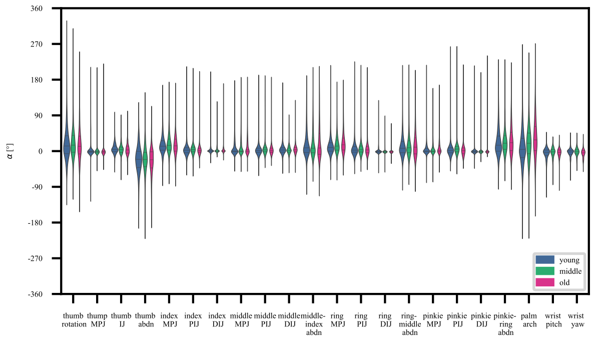

(a) Category A

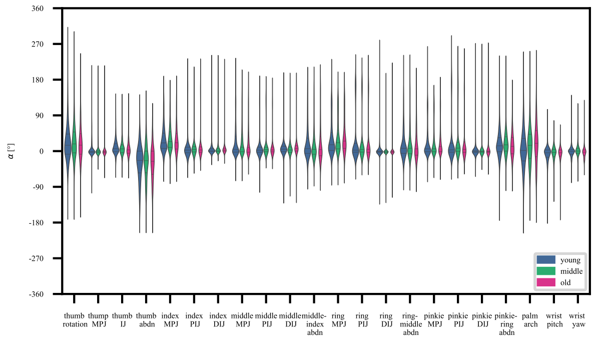

(b) Category B

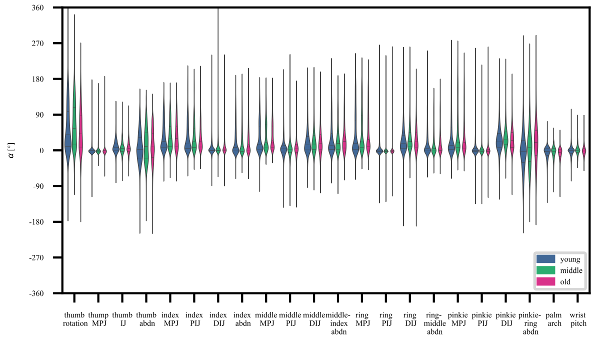

(c) Category C

Figure S5. Violin plots depicting joint angle distributions prior to re-calibration over 22 sensors for the 3 movement categories, split by age group. Quartile Positions and Median Angles of exercises are shown with dashed lines.

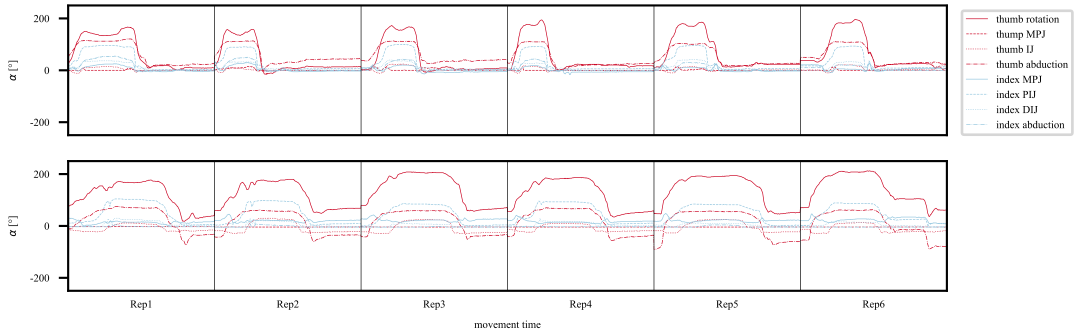

Figure S6. Exemplary concatenated angle trajectories for a large diameter grasp movement (actionID C1) of data that was only offset-calibrated. The data shown here were recorded from the thumb and index finger sensors (see legend for details). In the recordings one young participant (top panel) and one old participant (bottom panel) performed all six movement repetitions (Rep; separated by vertical lines).

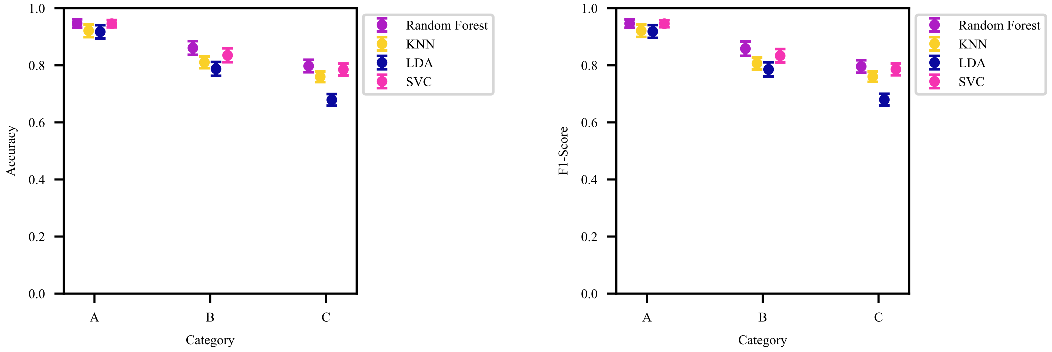

(a) Classification accuracy

(b) Classification F1-score

Figure S7. Mean accuracy and F1 scores as performance evaluation of the four employed classifiers in classifying the three movement categories on the offset-calibrated data. Symbols represent the mean values and whiskers the standard deviations.

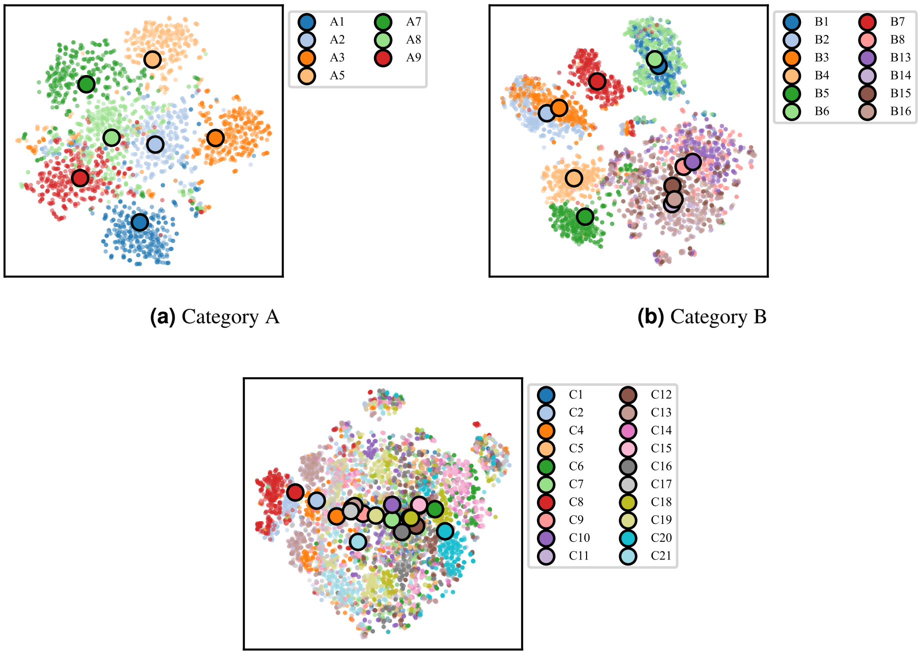

(a) Category A

(b) Category B

(c) Category C

Figure S8. t-SNE embeddings of raw sensor data (offet-calibrated only) employed on the three different exercise categories. Scatters represent individual embeddings and larger circles indicate actionID centroids.

References

1. Muschter, E. *et al.* Coming in handy: CeTI-Age — a comprehensive Database of kinematic Hand Movements across the Lifespan. Datasets. *Figshare* https://doi.org/10.6084/m9.figshare.c.6688871 (2023).
2. Cyberglove Systems LLC. *CGIII Manuals*. Cyberglove Systems LLC, http://www.cyberglovesystems.com (2010).
